# SarZ inhibits the hemolytic activity through regulation of phenol soluble modulins in *Staphylococcus epidermidis*

**DOI:** 10.3389/fcimb.2024.1476287

**Published:** 2024-11-19

**Authors:** Xiao Chen, Huiru Sun, Wei Wang, Han Wang, Runan Tan, Tao Zhu

**Affiliations:** ^1^ Department of Medical Microbiology and Immunology, Wannan Medical College, Wuhu, China; ^2^ Department of Pharmacy, Wannan Medical College, Wuhu, China

**Keywords:** *Staphylococcus epidermidis*, sarZ, hemolysis, phenol-soluble modulins, biofilm

## Abstract

**Background:**

*Staphylococcus epidermidis* is an important conditionally pathogenic bacterium. SarZ, belonging to the SarA family protein, has been demonstrated in *S. aureus* to promote the expression of invasive virulence factors while inhibiting biofilm formation. However, the regulatory role of SarZ on *S. epidermidis* virulence is not completely understood.

**Results:**

In this study, we successfully deleted the *sarZ* gene by allelic replacement in *S. epidermidis*. The *sarZ* mutant strain exhibited remarkably increased hemolytic activity and drastically impaired biofilm formation, suggesting that SarZ is key regulator of virulence in *S. epidermidis*. Through butanol extraction of the spent medium and HPLC-MS/MS analysis, production of phenol soluble modulins (PSMs) possessing cytolytic effect was found to be elevated significantly in the mutant. Subsequent qRT-PCR experiments demonstrated that expression of the *psm* genes, especially the β-type, was upregulated dramatically in the mutant. Meanwhile, transcription of *icaA* gene responsible for biofilm formation was sharply diminished. The *sarZ psmβ* double mutant was further generated and displayed a significantly decreased hemolytic activity compared with the *sarZ* mutant. EMSA assays implied that recombinant SarZ protein can directly bind to the promoter regions of the *psmβ* and *ica* operon. DNase I footprinting assays further pinpointed two SarZ-binding sites on the *psmβ* operon promoter.

**Conclusion:**

Taken together, the results confirmed that SarZ is a pivotal regulator of virulence in *S. epidermidis* and might respectively regulate the hemolytic activity and biofilm formation mainly by directly controlling the transcription of *psm* genes, particularly the β-type, and the *ica* operon.

## Introduction

*S. epidermidis* is considered as an ‘accidental pathogen’ (Otto, 2009; Becker et al., 2014; Severn and Horswill, 2023), since it can cause nosocomial infections and indwelling medical device-associated infections (Otto, 2013; Raad et al., 1998; Karchmer et al., 1983). The major pathogenesis of those opportunistic infections is attributed to the colonization of *S. epidermidis* on both biotic and abiotic surfaces, and subsequent formation of structured multicellular communities known as biofilm. Biofilm can render bacteria embedded within a self-produced extracellular matrix more resistant to attacks by host defenses and antibiotics (Costerton et al., 1999; Stewart and Costerton, 2001; Otto, 2018). In most cases, biofilm formation of *S. epidermidis* requires the polysaccharide intercellular adhesin, which is synthesized by the *ica* locus-encoded enzymes (Heilmann et al., 1996; Fluckiger et al., 2005). The *ica* locus comprises one regulator gene and four structural genes (*icaA*, *icaD*, *icaB* and *icaC*) that are organized in an operon (Cue et al., 2012). The *icaR* gene is diverently transcribed from the *icaADBC* operon and encodes a transcriptional repressor of the operon (Cerca et al., 2008).

In addition, *S. epidermidis* can also secrete exotoxins (peptides) and proteases (Lai et al., 2007; Peschel and Otto, 2013). For instance, phenol-soluble modulins (PSMs) are a class of short, amphipathic, α-helical peptides, which were first discovered in *S. epidermidis* with pro-inflammatory activity and biofilm-inhibitory property, and then found to have strong cytolytic effects toward leukocytes and erythrocytes in *S. aureus* (Otto, 2013, 2018; Mehlin et al., 1999; Cheung et al., 2012; Kreutzberger et al., 2022). According to their length, PSMs can be classified into the shorter α-type (approximately 20 to 25 amino acids long), and the longer β-type (40 to 45 amino acids long) (Peschel and Otto, 2013).

As we know, expression of those virulence determinants above are affected by environmental cues and coordinated by an array of regulatory proteins (Arvidson and Tegmark, 2001). It is worth noting that the SarA protein family, a collection of DNA binding proteins homologous to SarA, are adopted by the *Staphylococcus* genus to regulate virulence gene expression in response to changing microenvironment (Ballal et al., 2009). The common structural feature of the family is the presence of winged helix motif important for DNA binding and function (Kaito et al., 2006). Until now, eleven members have been identified in the family, which can be further divided into three subfamilies based on sequence and structural variation (Cheung et al., 2008).

Among them, SarZ and MgrA are classified as MarR subfamily due to their more sequence similarity to the MarR protein of Gram-negative bacteria than to the other SarA homologs (Cheung et al., 2008; Ballal et al., 2009). In *S. aureus*, SarZ has been extensively studied and shown to promote the expression of virulence factors such as hemolysin while inhibiting biofilm formation (Kaito et al., 2006; Tamber and Cheung, 2009). Transcriptional profiling revealed the *sarZ* regulon consists of genes involved in metabolic switching, antibiotic resistance, oxidation resistance, virulence, and cell wall properties (Chen et al., 2009; Lei and Lee, 2022; Dyzenhaus et al., 2023). Further gel shift assays demonstrated that SarZ protein uses a key Cys residue to sense oxidative stress and coordinate expression of its regulon (Liu et al., 2013). Whereas in *S. epidermidis*, just an insertional mutant strain of the *sarZ* gene were constructed by transposon mutagenesis in the clinical isolate 1457 (Wang et al., 2008), and then it was observed that the mutant strain displayed increased hemolytic activity and impaired biofilm formation, indicating that SarZ also acts as a key regulator of virulence in *S. epidermidis*, but probably in a manner opposite to its ortholog in *S. aureus* (Kaito et al., 2006; Wang et al., 2008). Therefore, the exact regulatory effect of SarZ on the expression of virulence factors in *S. epidermidis* and the underlying molecular mechanism remains elucidated.

In this study, through construction of a *sarZ* knockout mutant in the clinical isolate RP62A, we demonstrated that SarZ can suppress hemolytic activity and enhance biofilm formation in *S. epidermidis*. Moreover, we revealed that the regulation of hemolytic capacity by SarZ is accomplished by controlling the production of PSMs, especially the β-type. Further analysis indicated that SarZ can govern the transcription of the *psmβ and ica* operons by directly binding to their promoters. Taken together, these results showed that SarZ divergently modulates the expression of virulence factors by exerting its effect as a transcriptional factor.

## Materials and methods

### Strains, plasmids and growth conditions

The bacterial strains and plasmids used in this study are listed in **Table 1**. The *S. epidermidis* wild-type (WT) strain and its isogenic mutants were routinely grown in Tryptic soy broth (TSB) or Brain Heart Infusion (BHI) with a shaking speed of 220 rpm at 37°C, and *E. coli* strains were cultured in Lysogeny Broth (LB). When necessary, antibiotics were added to the above media to the following final concentrations: 50 μg·ml^-1^ carbenicillin and 10 μg·ml^-1^ chloramphenicol.

**Table 1:** Bacterial strains and plasmids used in this study.

Strain/Plasmid	Relevant characteristics ^a^	Sources or reference
Strain		
RP62A	Methicillin-resistant, biofilm-forming *Staphylococcus epidermidis* isolate; genome sequenced	(Gill et al., 2005)
*E. coli* DC10B	dam*^+^* dcm*⁻ ΔhsdRMS* endA1 recA1	(Monk et al., 2012)
*ΔsarZ*	a *sarZ* deletion mutant of RP62A	This study
*ΔsarZ psmβ*	a *sarZ psmβ* double deletion mutant of RP62A	This study
C- *psmβ*	*sarZ psmβ* double mutant complemented with pCNcat-psmβ	This study
C-*sarZ*	*sarZ* mutant complemented with pCNcat*-sarZ*	This study
C-pCN	*sarZ* mutant complemented with pCNcat	This study
BL21 star (DE3)	F- *omp*T *hsd*S_B_ (r_B_⁻ m_B_⁻) *gal dcm rne*131 (DE3)	Shanghai Beyotime Biotech
Plasmid		
pUCm-T	Using for T/A cloning	Shanghai Sangon Biotech
pUCm-T-*sarZ*	pUCm-T containing 776-bp upstream and 912-bp downstream fragments of *sarZ*	This study
pUCm-T-*psmβ*	pUCm-T containing 1015-bp upstream and 1037-bp downstream fragments of *psmβ*	This study
pKOR1	Temperature-sensitive shuttle vector for allelic exchange in *S. epidermidis*; Amp^r^ Cm^r^	(Bae and Schneewind, 2006)
pKOR1-*ΔsarZ*	pKOR1 containing 776-bp upstream and 912-bp downstream fragments of *sarZ*	This study
pKOR1-*Δpsmβ*	pKOR1 containing 1015-bp upstream and 1037-bp downstream fragments of *psmβ*	This study
pCN51	*E. coli*-*Staphylococcus* shuttle vector; Amp^r^ Em^r^ (*ermC*)	(Charpentier et al., 2004)
pCNcat	pCN51, where *ermC* is replaced by *cat194* for complementation	This study
pCNcat-*sarZ*	pCNcat containing the full-length *sarZ* gene and its promoter region *sarZ*	This study
pCNcat-*psmβ*	pCNcat containing the full-length *psmβ* gene and its promoter region	This study
pET28a	*E. coli* expression plasmid; Km^r^	(Wu et al., 2015)
pET28a-*sarZ*	pET28a harboring the *sarZ* gene, used for recombinant expression of SarZ	This study

^a^ Km^r^, kanamycin resistance; Amp^r^, ampicillin resistance; Cm^r^, chloramphenicol resistance; Em^r^, erythromycin resistance.

### Construction of the *sarZ* mutant and the *sarZ psmβ* double mutant strains

The primers used in this study are listed in **Supplementary Table 1**. For construction of the *sarZ* knockout mutant, upstream and downstream DNA fragments flanking of the *sarZ* gene were PCR amplified from the genomic DNA of *S. epidermidis* strain RP62A.The two DNA fragments were further combined by fusion PCR and then inserted into the pUCm-T vector through TA cloning. The resulting vector was taken as the template to obtain the two homology arms with restriction endonuclease sites Apa I and Nco I by PCR amplification. The homologous arms were then cloned into *E. coli*-*staphylococcal* shuttle vector pKOR1 by using conventional molecular cloning technique, yielding the recombinant plasmid pKOR1-*ΔsarZ*. The plasmid was transformed into *E. coli* strain DC10B for restriction modification and subsequently electroporated into *S. epidermidis* strain RP62A. For construction of the *sarZ psmβ* double mutant, the two homology arms flanking the entire *psmβ* operon were obtained by adopting the cloning strategy mentioned above. Instead, the recombinant plasmid pKOR1-*Δpsmβ* were generated through In-Fusion cloning according to the manual of ClonExpress Ultra One Step Cloning Kit (Vazyme, Nanjing, China). The remaining steps for allelic replacement were performed as described previously (Zhu et al., 2017). The desired mutant strains were screened by PCR with a pair of primers complementary to regions outside the homology arms and eventually confirmed by DNA sequencing.

### Complementation of the *sarZ* mutant and the *sarZ psmβ* double mutant strains

For complementation of the *sarZ* mutant strain, a DNA fragment encompassing the *sarZ* gene and its promoter region was amplified, and then ligated into pCNcat to create pCNcat-*sarZ* through In-Fusion cloning. For complementation of the double mutant strain, a DNA fragment containing the entire *psmβ* operon and its promoter sequences was cloned into pCNcat as described above. The promoter sequences were predicted by using BDGP Neural Network Promoter Prediction software (http://www.fruitfly.org/seq tools/).

### Hemolytic activity assay

To detect the hemolytic activity of *S. epidermidis* wild-type and its isogenic mutants, all the strains were cultivated to exponential phase in BHI medium. Then, a 2.5 μl aliquot of each culture was withdrawn and spotted onto Columbia blood agar plate. The plates were incubated at 37°C for approximately 24 hours and the hemolytic zones were examined and photographed.

### Biofilm production assay

To investigate the effect of *sarZ* mutation on *S. epidermidis* biofilm formation, a semiquantitative microplate assay involving crystal violet staining was performed as described previously (Zhu et al., 2017).

### Biofilms observed by SEM

Differential biofilm formation was further observed using scanning electron microscopy (SEM). Briefly, *S. epidermidis* cells were seeded into a 6-well tissue culture plate containing segments of central venous catheter, and grown in TSB medium at 37°C for 24 hours. After that, catheter segments were washed three times with 1×PBS to remove planktonic bacteria and fixed with 2.5% glutaric glutaraldehyde at 4°C for 6 hours. After progressive alcohol dehydration, catheter segments were air-dried, mounted onto the SEM holder with black glue and gold-sputtered. SEM micrographs were taken at ×3000 and ×10000 magnification.

### Isolation of crude phenol-soluble modulins

Crude PSMs were extracted from *S. epidermidis* according to the protocol reported by Joo et al. (Joo and Otto, 2014) with some modifications. Briefly, *S. epidermidis* cells were cultured overnight in BHI medium, and subsequently centrifuged to collect the supernatant. The supernatant was clarified by filtration, and then added with 1/3 of 100% 1-butanol to make 25% of 1-butanol. Then, the mixture was shaken vigorously (260 rpm) at 37°C for 2 hours. After brief centrifugation, the upper phase (1-butanol phase) was harvested and then evaporated using conventional rotary evaporation at 60°C. To accelerate the evaporation process, the distilled water was added into the 1-butanol phase (1-butanol phase: distilled water=1:2 (v/v)). Finally, the precipitates were redissolved in distilled water to acquire the crude PSMs and then visualized by 12% SDS-PAGE.

Hemolytic activity of the crude PSMs was determined with sheep red blood cells. One hundred microliters of crude PSMs extracts were mixed with 900 μl of 1×PBS buffer containing 3% sheep red blood cells. The mixtures were then incubated at 37°C for 2 hours. After centrifugation, the absorbance of the supernatant was measured at 540 nm to quantify the released hemoglobin in order to evaluate the extent of erythrocyte lysis.

### HPLC-MS/MS

After reduced by 10 mM DTT at 56°C for 1 hours and alkylated by 50 mM IAM at room temperature in dark for 40 min, the crude PSMs were lyophilized to near dryness, and then resuspended in 0.1% formic acid. HPLC-MS/MS experiments were carried out on an Easy-nLC 1000 system connected to an Orbitrap Exploris™ 240 Mass Spectrometer (Thermo Fisher Scientific, USA) equipped with an ESI nanospray source. Chromatographic separation was performed on an in-house made NanoColumn (15cm, ID 150 μm, 3μm, C18) with a flow rate of 600 nL/min. The LC linear gradient was used from 6% to 9% B for 5 min, from 9% to 14% B for 15 min, from 14% to 30% B for 30 min, from 30% to 40% B for 8 min and from 40% to 95% B for 2 min. Solvent A was 0.1% formic acid in water, and solvent B was 0.1% formic acid in acetonitrile/water (80:20, v/v). For ionization, a spray voltage of 2.2 kV and a 320°C capillary temperature was used. Peptides were analyzed in positive mode from 350 to 1,500 m/z, followed by data-dependent higher-energy collision dissociation (HCD) MS/MS scans using a normalized collision energy of 30%.

The raw MS files were analyzed and searched against the UniProt Staphylococcus protein databases using Byonic. The parameters were set as follows: the protein modifications were carbamidomethylation (C) (fixed), oxidation (M) (variable), Acetyl (Peptide N-term) (variable), Formyl (Peptide N-term) (+27.99) (variable); the enzyme specificity was set to trypsin; the maximum missed cleavages were set to 3; the precursor ion mass tolerance was set to 20 ppm, and MS/MS tolerance was 0.02 Da. Only high confident identified peptides were chosen for downstream analysis. The relative quantification of PSMs peptides were performed by integration of extracted ion chromatograms of formylated and deformylated forms.

### Total RNA extraction, cDNA synthesis, and real time PCR

*S. epidermidis* cells were grown in six milliliters of BHI medium at a flask-to-media volume ratio of 5:1 to the mid-logarithmic phase (4 hours) and stationary phase (10 hours), respectively. Total RNA was prepared using the RNeasy-mini kit (Qiagen) according to the manufacturer’s instructions with some modifications as described before (Zhu et al., 2022). Afterward, 0.5 μg of total RNA was taken to synthesize cDNA using the Go Script™ Reverse Transcription Mix (Promega). The qPCR reactions were run on a LightCycler^®^ 96 instrument. Fast qPCR Master Mix (2×) (Roche) was used along with specific primers as listed in **Supplementary Table 1**. Each reaction was carried out in triplicate, and the *gyrB* gene was employed as reference gene for normalization. The relative quantity of target genes was calculated using the 2^-ΔΔct^ method.

### Expression and purification of recombinant SarZ protein

The *sarZ* gene was amplified with the primers in **Supplementary Table 1** and inserted into the vector pET28a to construct a recombinant expression plasmid pET28a-*sarZ*. The resulting plasmid was then transferred into *E. coli* BL21 star (DE3). The expression strain was grown in LB medium with 50 μg·ml^-1^ kanamycin at 37°C, shaking at 220 rpm until OD_600_ was 0.6, and then added with a final concentration of 1 mM isopropyl β-D-thiogalactopyranoside (IPTG) at 37°C for additional 4 hours’ cultivation. The culture was harvested by centrifugation, and the pellet was resuspended in lysis buffer (20 mM Na_2_HPO_4_, 500 mM NaCl, 20 mM imidazole, pH 7.4) and then homogenized on ice by sonication. After centrifugation, the supernatant was collected and purified through Ni-IDA sefinose Resin (Shanghai Sangon Biotech). The His-tagged SarZ protein was eluted with 20, 100, 150, 300 mM imidazole. The purity of the recombinant protein was checked by 12% SDS-PAGE. The protein concentration was determined using a Bradford protein assay kit.

### Electrophoretic mobility shift assay

For EMSA assay, the 5’-biotin-labeled DNA fragments containing the promoter regions of the *psms* and *ica* operon were amplified from the *S. epidermidis* genomic DNA using the primers listed in **Supplementary Table 1**. Then, the biotin-labeled DNA fragments were incubated with increasing concentrations of purified SarZ protein at room temperature for 20 min in binding buffer (Shanghai Beyotime Biotech). For competitive EMSA, a 100-fold and 200-fold molar excess of unlabeled fragment was respectively added to the reaction mixture for 20 min prior to addition of a constant amount of labeled fragment. After incubation, the mixtures were electrophoresed on a 5% native polyacrylamide gel in 0.5× TBE buffer and then the gel was blotted onto a positively charged nylon membrane. The shifted bands were detected and analyzed using the LightShift Chemiluminescent EMSA kit (Shanghai Beyotime Biotech). The *rpsJ* (encoding 30S ribosomal protein S10) gene was designated as negative control for SarZ-DNA binding.

### DNase I footprinting assay

A 330-bp fragment (from 735785 bp to 736114 bp), covering the 276-bp of *psmβ* promoter region in the EMSA assay, was synthesized and then ligated into pUC57 vector through in-fusion cloning. The promoter fragment was fluorescently labeled by PCR amplification with the primers listed in **Supplementary Table 1**. Footprinting assays were performed according to the method described previously (Queck et al., 2008), with some modifications. Briefly, binding reactions were set up in 40 μl volumes, which contained 1×binding buffer (Shanghai Sangon Biotech), 50 ng/μl salmon sperm DNA, 500ng FAM-labeled DNA fragment and various amounts of purified SarZ. After incubation at room temperature for 30 min, 5 microliters of enzyme mixture comprising 1× RQI buffer (Shanghai Sangon Biotech), 10 mM CaCl_2_, 0.1U/μl DNase I (Thermo Fisher) was added. The DNase I digestion was carried out for about 55 s and terminated by adding 10 μl of 0.5 M EDTA. Final DNA fragments were extracted using DiaSpin PCR Product Purification Kit (Shanghai Sangon Biotech) and detected with an Applied Biosystems 3730XL DNA analyzer. Electropherograms were analyzed and aligned using the GeneMapper software (Applied Biosystems). The assay was repeated at least three times with similar results.

### Statistical analyses

Experimental data obtained were analyzed using GraphPad Prism8 and compared by the independent-sample t-test or one-way analysis of variance (ANOVA). P< 0.05 were considered statistically significant.

## Results

### Deletion of *sarZ* gene increased hemolytic activity of *S. epidermidis*

**Figure 1:**
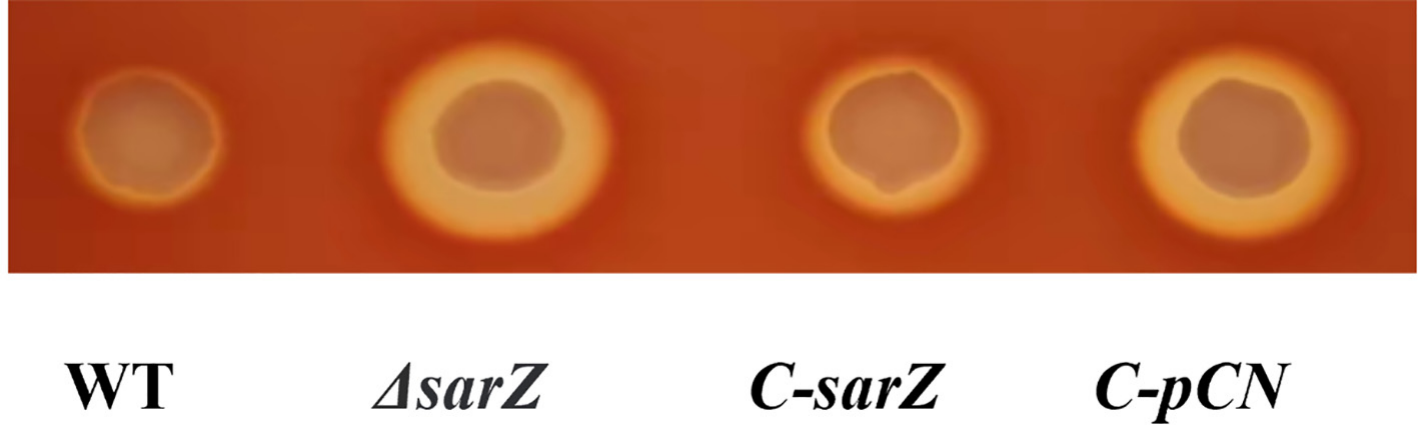
Deletion of *sarZ* in *S. epidermidis* led to increased hemolytic activity. The hemolytic zones of the WT, *ΔsarZ*, *C-sarZ* and *C-pCN* strains were observed by spotting culture aliquots onto Columbia blood agar plates for overnight incubation. WT, the wild type strain; *ΔsarZ*, the *sarZ* mutant; C-*sarZ*, the *sarZ* mutant complemented with the native *sarZ* gene; *C-pCN*, the *sarZ* mutant complemented with the empty vector *pCNcat*.

To confirm the regulatory role of SarZ on virulence factors in *S. epidermidis*, the *sarZ* deletion mutant strain was successfully constructed via homologous recombination (**Supplementary Figures 1**–**4**). It was found that the *sarZ* mutant strain displayed a significantly larger zone of hemolysis than the WT strain when aliquots of both strains were spotted onto Columbia blood agar plate and then incubated at 37 °C for 24 hours. Complementation of the *sarZ* mutant strain by pCNcat-*sarZ* restored the hemolytic zone at size close to the WT strain, whereas empty vector had no effect on the hemolysis (**Figure 1**). These results indicated that SarZ inhibits the hemolytic activity of *S. epidermidis*.

### Deletion of *sarZ* gene decreased biofilm formation of *S. epidermidis*

**Figure 2:**
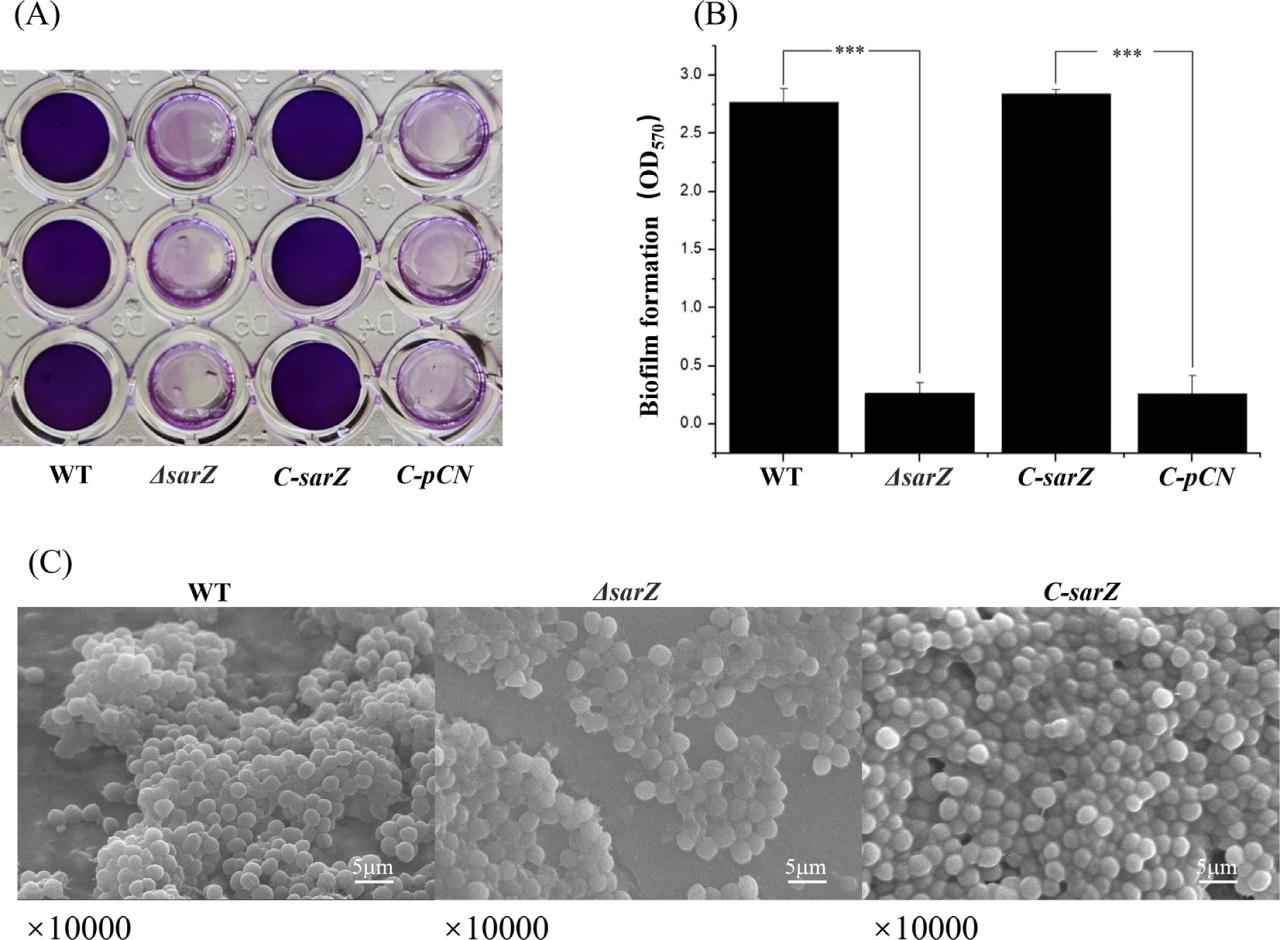
Deletion of *sarZ* led to decreased biofilm formation in *S. epidermidis*. A static biofilm formation assay was performed in 96-well polystyrene plates. *S. epidermidis* strains were grown at 37°C in TSB medium. Mature biofilms (24 hours) were visualized using crystal violet staining **(A)** and then semi-quantified by measuring the absorbance at 570 nm **(B)**. All experiments were performed in triplicate. The data were represented as means ± the SEM. ***P<0.001. Scanning electron micrographs of biofilms formed by *S. epidermidis* on catheter **(C)**.

To investigate whether SarZ influences the biofilm formation of *S. epidermidis*, a semiquantitative biofilm assay was carried out. As shown in **Figures 2A, B**, the *sarZ* mutant strain formed significantly reduced biofilm compared to the WT strain. The amount of biofilm was restored to the wild-type level after complementation of the *sarZ* mutant strain. Furthermore, the SEM images showed that the WT strain generated a dense biofilm comprising multiple layers of bacterial cells on the catheter surface, whereas the *sarZ* mutant strain just formed a few single-layer bacterial clusters (**Figure 2C**). Altogether, these findings confirmed that SarZ promotes biofilm formation in *S. epidermidis*.

### The amount of PSMs was elevated in the *sarZ* mutant strain

**Figure 3:**
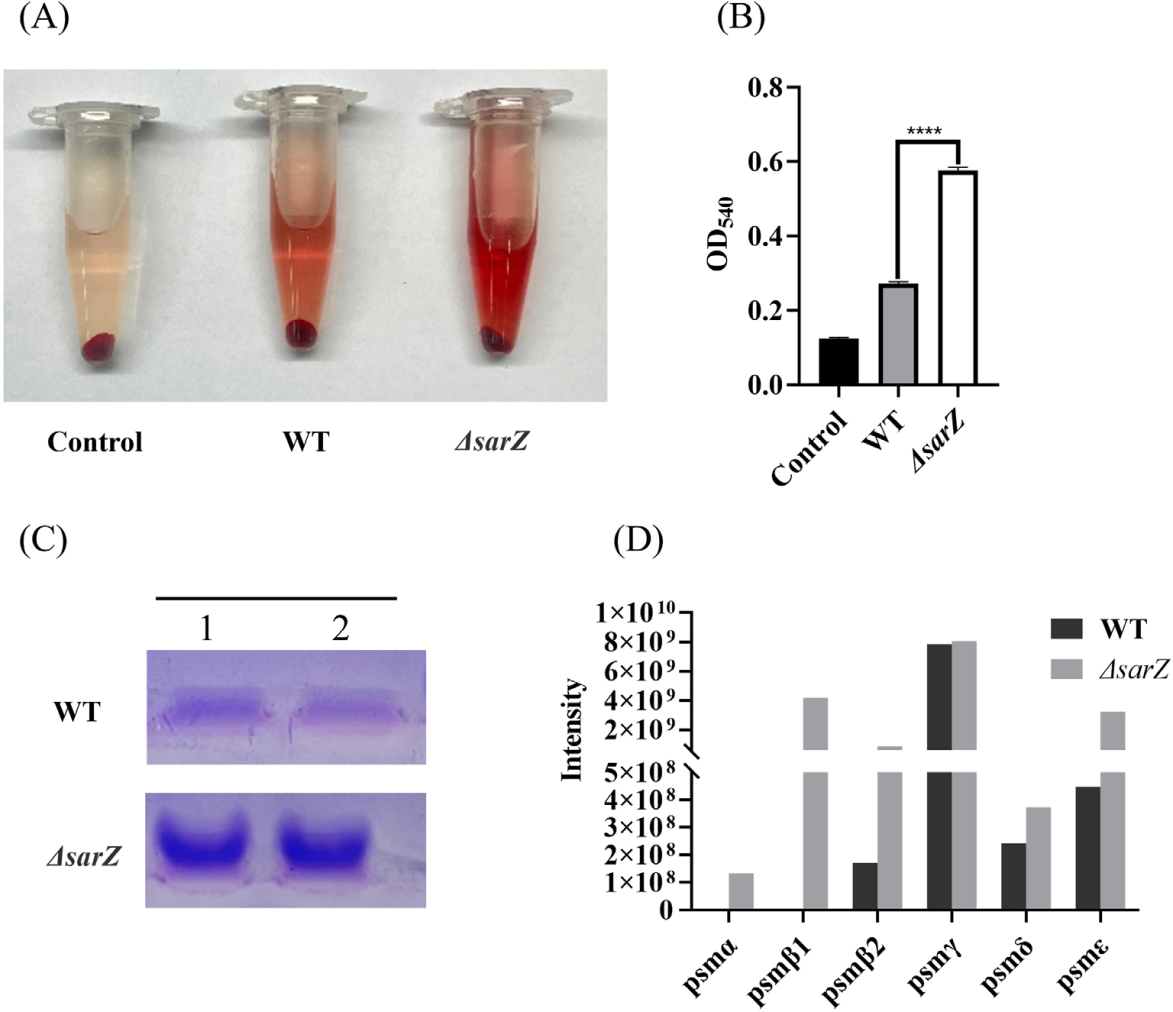
Qualitative and relative quantitative analysis of the crude PSMs. The crude PSMs peptide were prepared from the *ΔsarZ* and WT strain by n-butanol extraction method. Hemolytic activities were determined by incubating the crude PSMs with 3% sheep red blood cell. PBS was used as a negative control **(A)**. The extent of lysis was measured by the amount of hemoglobin based on its absorption at 540 nm **(B)**. The crude PSMs peptides were visualized using the Coomassie blue stained 12% SDS-PAGE **(C)**. All the above experiments were performed in triplicate. The data are represented as means ± the SEM. Statistical significance was determined by the one-way analysis of variance (ANOVA). ****P<0.0001. PSMs production was measured by HPLC-MS/MS and quantified by the sum of extracted ion chromatograms of formylated and deformylated forms **(D)**.

Since PSMs in *Staphylococcus* were reported to have cytolytic capacity toward erythrocytes and neutrophils during the past decade, it was speculated that SarZ might regulate the hemolytic activity by inhibiting the production of PSMs. To test the hypothesis, crude PSMs were isolated from the spent medium of the *sarZ* mutant and its parent strain by 1-butanol extraction, and their hemolytic activities were further compared. As expected, butanol extract of the *sarZ* mutant strain exhibited significantly stronger cytolytic activity against sheep erythrocytes than the counterpart of the WT strain (**Figures 3A, B**). Furthermore, the crude PSMs were resolved by SDS-PAGE. Compared with the WT strain, the protein band corresponding to PSMs were obviously more intensive in the *sarZ* mutant strain (**Figure 3C**), indicating that PSMs production was significantly higher in the *sarZ* mutant strain than in the WT strain.

Subsequently, in order to identify the PSMs member whose production is primarily affected by *SarZ*, HPLC-MS/MS was performed to distinguish the differences in PSMs content between the *sarZ* mutant strain and its parent strain. A total of six PSMs that were previously found to be produced by *S. epidermidis*, including PSMα, PSMβ1, PSMβ2, PSMγ, PSMδ and PSMε, were detected. Among them, PSMβ1 and PSMα were only detected in the *sarZ* mutant strain, whereas no or little difference in PSMγ and PSMδ production was observed, suggesting that amounts of PSMβ1 and PSMα in the mutant strain were considerably higher than that in the parent strain (**Figure 3D**). In addition, PSMβ2 production was also obviously higher in the mutant strain. It is noteworthy that in both strains, the amount of β-type PSMs was more abundant than that of PSMα (**Figure 3D**), which is in accordance with previous study. These above results indicated that SarZ may inhibit the hemolytic activity of *S. epidermidis* by downregulating the production of the PSMs family, particularly the β-type PSMs.

### SarZ inhibits the expression of *psm* genes in *S. epidermidis*

**Figure 4:**
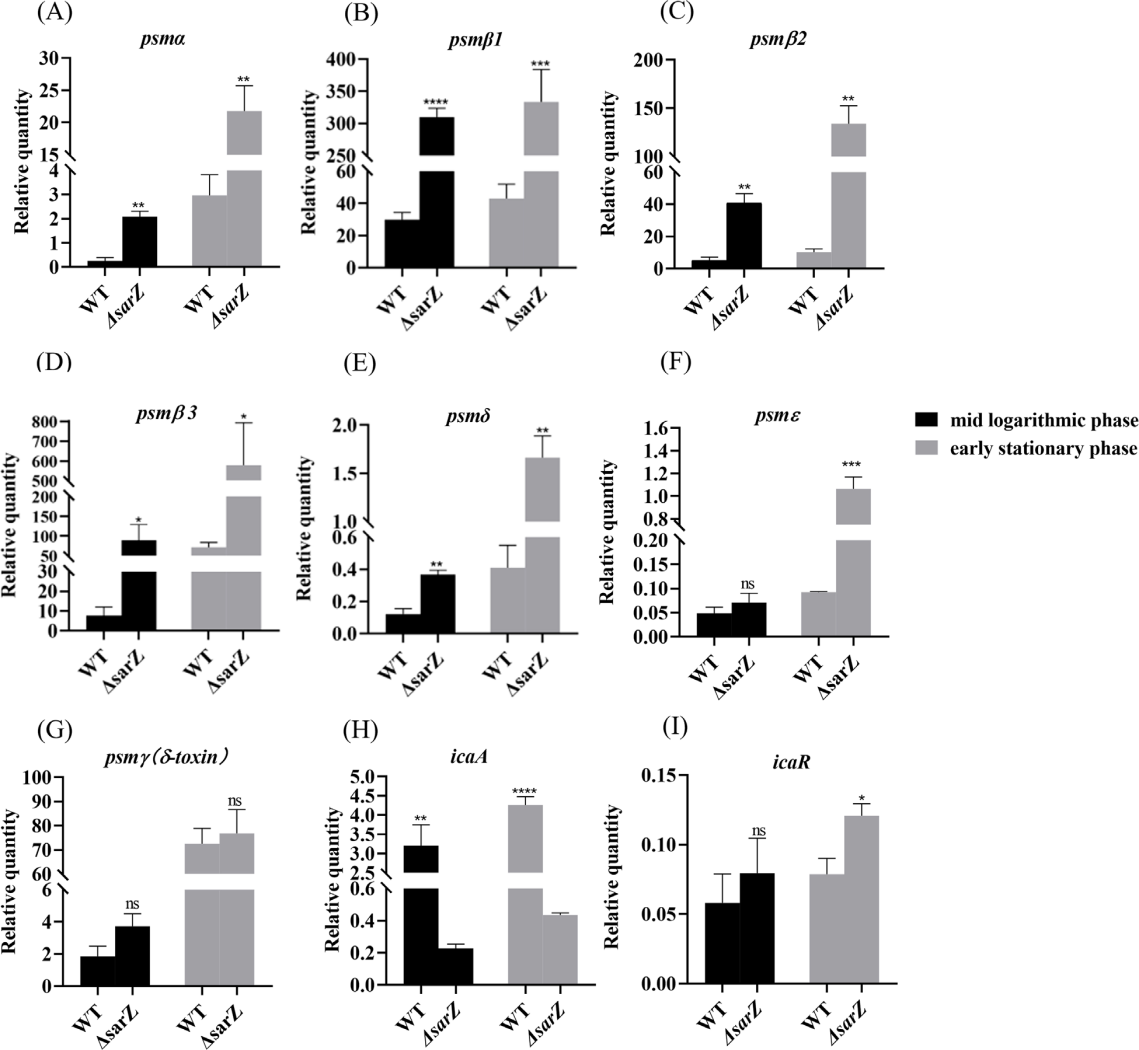
Disruption of *sarZ* resulted in the altered transcription of virulence genes. Real-time RT-PCR was employed to detect the relative expression of *psm* and *icaA* genes against the constitutively expressed *gyrB* gene in *S. epidermidis* RP62A and its *sarZ* mutant **(A–I)**. Data are expressed as the mean ± SEM of three independent experiments. Statistical significance was determined by the Student’s t test. *P<0.05; **P<0.01; ***P<0.001; ****P<0.0001. ns, not significant.

To confirm the substantial differences in the pattern of PSMs production between the *sarZ* mutant strain and the WT strain, transcript levels of all *psm* genes in both strains were measured using qRT-PCR. As a result, gene expression of *psmα , psmβ1*, *psmβ2*, *psmβ3*, *psmδ and psmε* was indeed upregulated obviously in the sarZ mutant strain (**Figures 4A–F**), which coincides with the MS data. In particular, the transcript levels of β-type *psm* were increased significantly, with a 10-, 8-, 11-fold increase in the logarithmic phase and 7-, 13-,8-fold increase in the early stationary phase for *psmβ1*, *β2* and *β3*, respectively (**Figures 4B–D**). Second, the transcript levels of *psm*α were also elevated markedly, with a respective 8- and 7-fold change in the logarithmic and early stationary phase (**Figure 4A**). As anticipated, the transcript levels of *psmγ, also known as hld*, did not differ between the wild type and *sarZ* mutant strain (**Figure 4G**). Consistent with the remarkably decreased biofilm formation, transcript level of *icaA* gene was notably downregulated in the *sarZ* mutant strain (**Figure 4H**). However, no difference was observed in the *icaR* transcript levels (**Figure 4I**). Taken together, these results demonstrated that SarZ inhibits transcription of *psm* genes, especially *psmβ* operon. Meanwhile, SarZ contributes to biofilm formation by activating transcription of *ica* operon independently of *icaR.*

### SarZ regulates the hemolytic activity of *S. epidermidis* through *psmβ*

**Figure 5:**
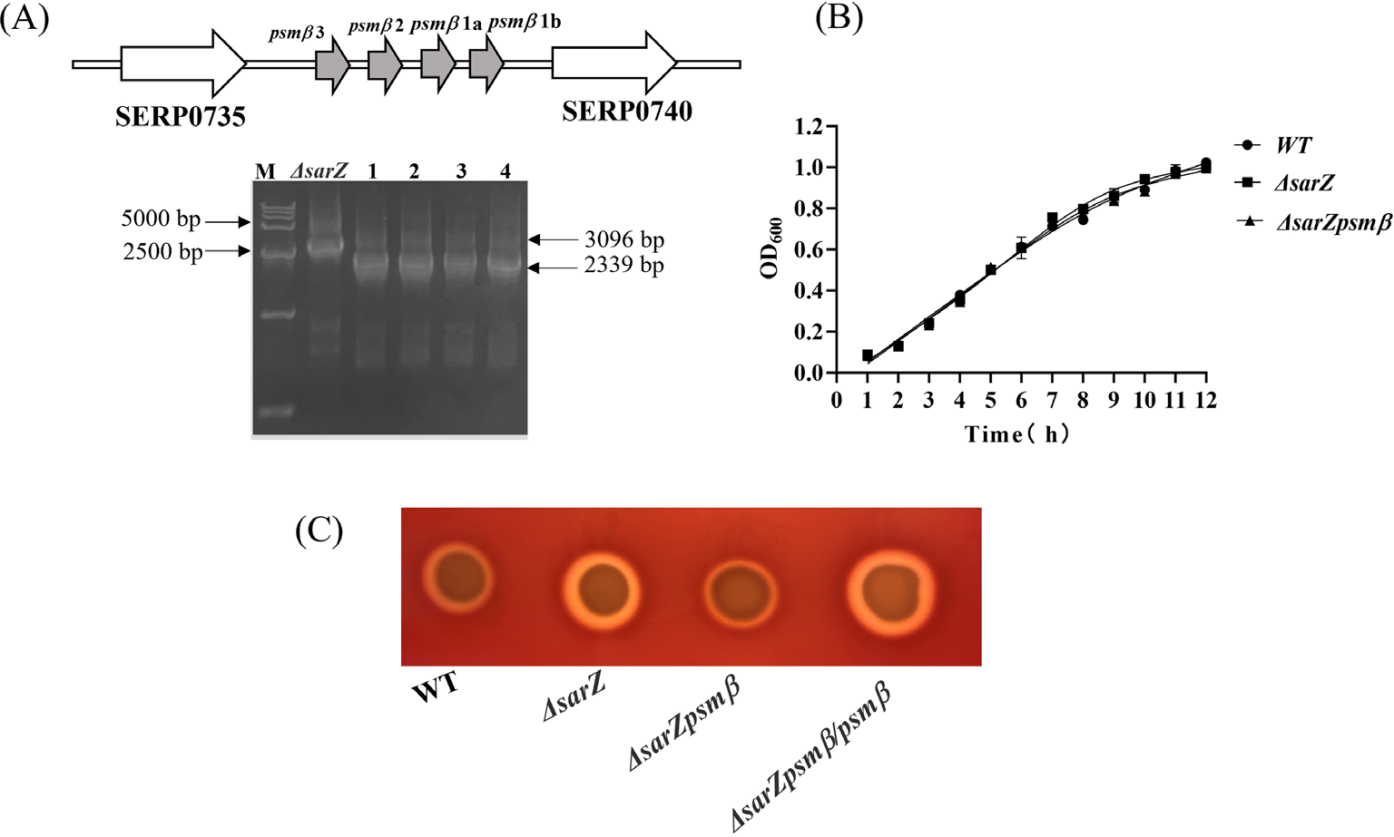
Construction and hemolytic activity of the *sarZ psmβ* double mutant strain. Schematic diagram of the composition of the *psmβ* operon and PCR identification of the *sarZ psmβ* double mutant. As indicated, the PCR product amplified from the double mutant was shorter in length than that from the single mutant, satisfied with the anticipated size after *psmβ* deletion **(A)**. A comparison of growth rates between the WT, *ΔsarZ* and *ΔsarZ psmβ* strains in TSB medium **(B)**. The hemolytic activities of the WT, *ΔsarZ*, *ΔsarZ psmβ* and *ΔsarZ psmβ/psmβ* (*ΔsarZ psmβ* complemented with the native *psmβ* gene) **(C)**.

Obviously increased expression of β-type *psm* revealed by both the HPLC-MS/MS and qRT-PCR in the *sarZ* mutant led us to speculate that SarZ may regulate the hemolytic activity of *S. epidermidis* mainly by controlling the expression of β-type *psm*. In order to verify our speculation, the *sarZ psmβ* double mutant strain was then constructed successfully by allelic replacement (**Supplementary Figures 5**–**7**; **Figure 5A**). No significant differences in the growth curves were monitored between the WT, *sarZ* mutant and *sarZ psmβ* double mutant strains (**Figure 5B**). As expected, the double mutant strain displayed a significant decreased hemolytic activity compared with the *sarZ* single mutant strain, reaching the level equivalent to the WT strain. The hemolytic zone was restored to the size comparable to the *sarZ* single mutant strain after complementation of the double mutant strain with the intact *psmβ* operon (**Figure 5C**). Taken together, these data demonstrated that the regulation of hemolytic activity by SarZ is dependent on the presence of *psmβ* operon in *S. epidermidis*.

### SarZ can bind to the promoter regions of the *psm* genes and *ica* operon

**Figure 6:**
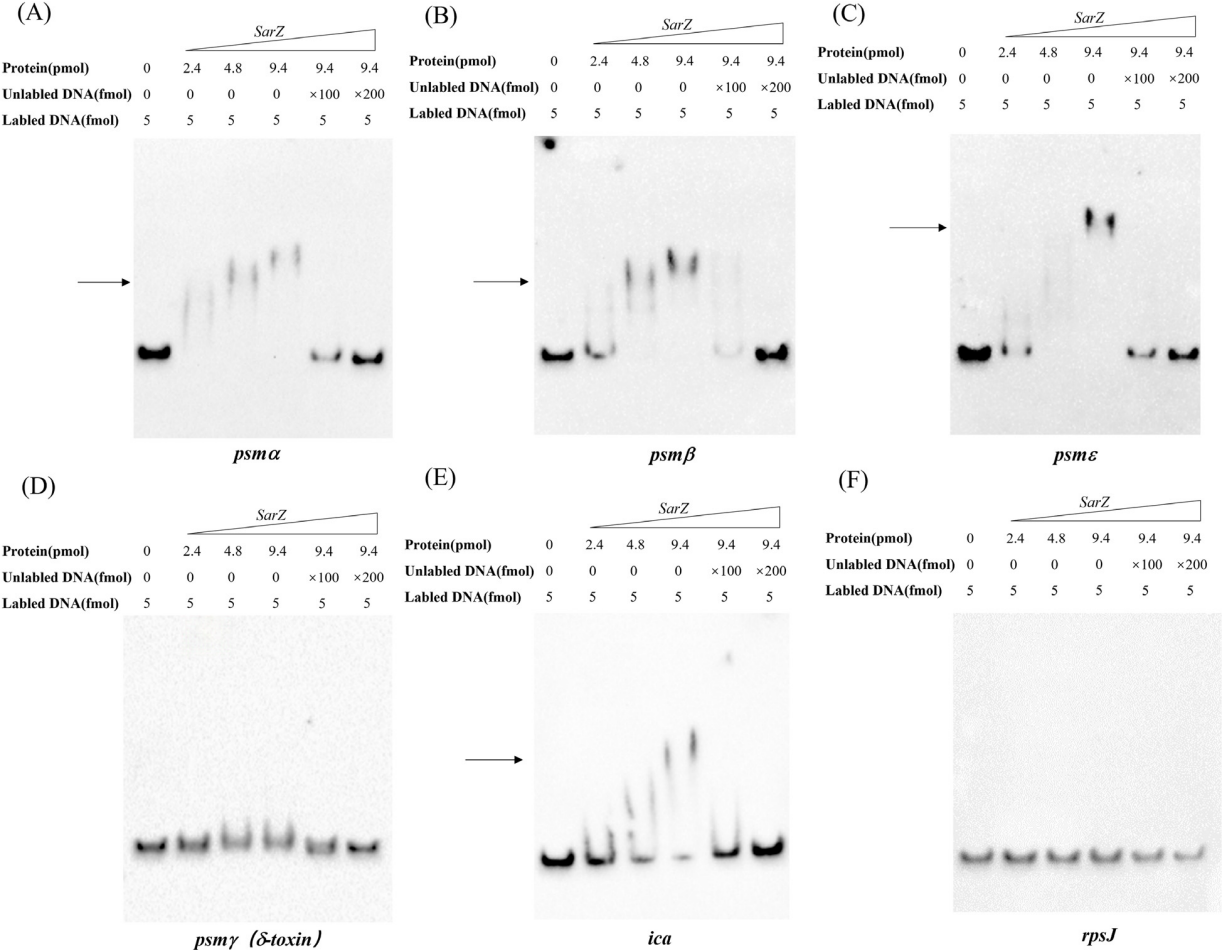
EMSA analysis of *S. epidermidis* SarZ with the promoter regions **(A–F)**. The promoter regions of *psm* genes and *ica* operon were labeled with biotin by PCR amplification. Gel shift reactions were performed by incubating labeled probe with increasing concentrations of SarZ (ranging from 2.4 to 9.4 pmol). Lane 1, 5 and 6 of each blot contained a no-protein control, a 100-fold and a 200-fold excess of unlabeled probe competitor control, respectively. All samples were electrophoresed on a 5% nondenaturing polyacrylamide gel and blotted onto nylon membrane. The arrows indicate the positions of SarZ-bound probes; The DNA fragment within the *rpsJ* coding region was used as a negative control.

As a member of the MarR-family proteins (Dyzenhaus et al., 2023), SarZ contains one predicted helix-turn-helix (HTH domain) DNA-binding domains. Since all *psm* genes transcription is altered in the *sarZ* mutant, the ability of SarZ binding directly to their promoter regions was investigated. The recombinant His-tagged SarZ was used for EMSA with biotin labeled DNA fragments containing the respective promoter regions of *psm* genes (262-bp *psmα*, 276-bp *psmβ*, 230-bp *psmε* and 183-bp *psmγ*). Despite repeated attempts, the promoter region of *psmδ* was always unable to be fluorescently labeled to conduct the assay. As shown in **Figure 6**, the promoter fragments of *psmα*, *psmβ* and *psmε* formed retarded DNA-protein complex with SarZ in a dose-dependent manner, respectively (**Figures 6A–C**, lane 2 to lane 4). The addition of a 200-fold excess of unlabeled identical DNA fragments as a specific competitor completely blocked SarZ-biotin-DNA complex formation (**Figures 6A–C**, lane 6). As expected, *psmγ* promoter fragment did not form a shifted complex with SarZ (**Figure 6D**). We also examined the ability of SarZ binding to the 165-bp labeled DNA fragment containing the *ica* operon promoter. As illustrated in **Figure 6E**, with the increase of SarZ dosage, the shifted band of *ica* operon promoter emerged and was strengthened. As a negative control, a 119-bp DNA fragment of *rpsJ* gene did not form a shifted complex with SarZ under the same condition (**Figure 6F**). Collectively, these data indicated that SarZ can specifically bind to the promoter region of the *psm* genes and *ica* operon, excluding the *psmγ*.

### Two SarZ binding sites were identified on the promoter of *psmβ* operons

**Figure 7:**
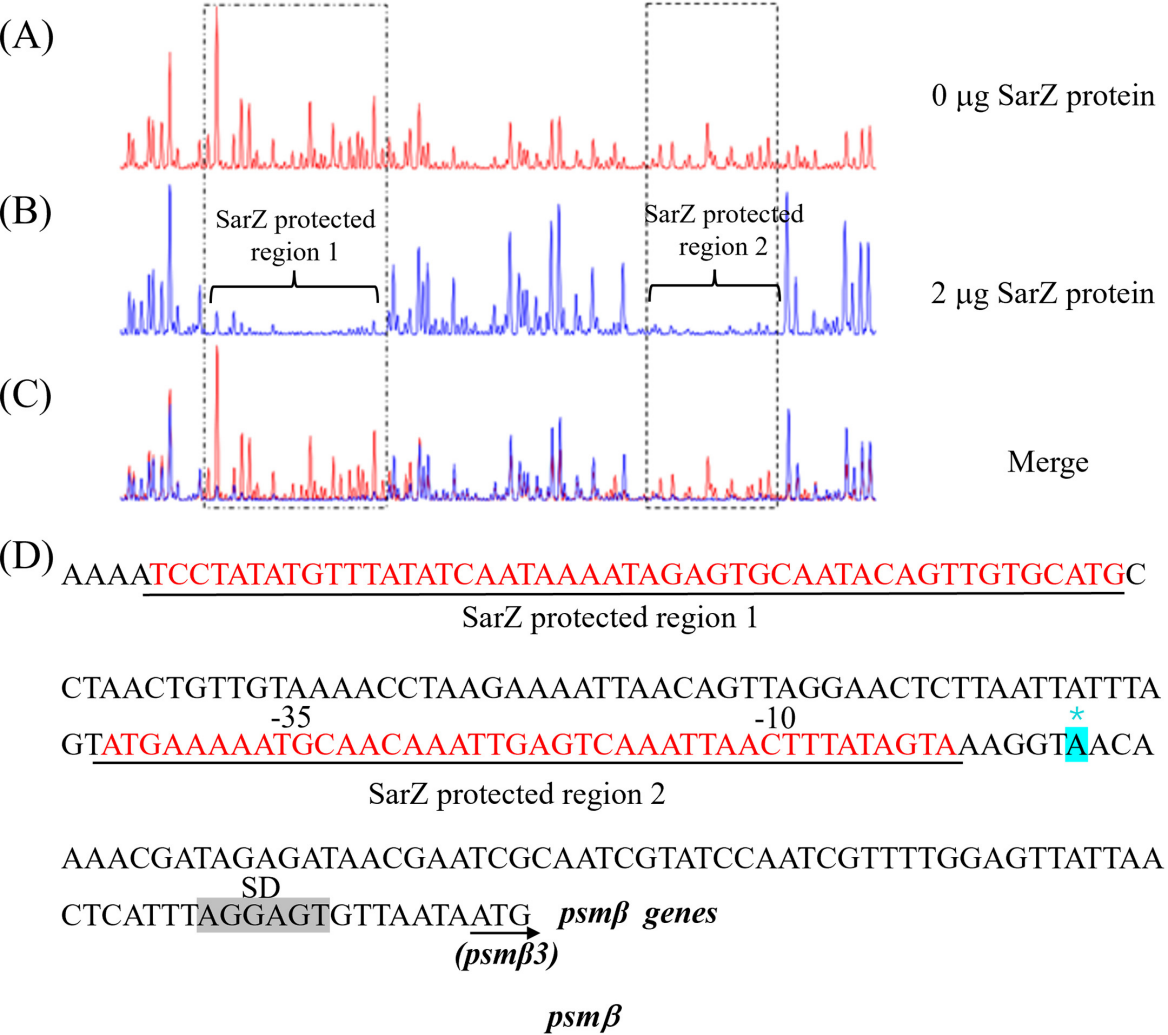
Mapping of the SarZ recognition site in the *psmβ* **(A–C)** promoter by DNase I footprinting. The promoter regions of *psmβ* was labeled with 6-FAM, and incubated with SarZ at 0 μg **(A)**, 2 μg **(B)** for 30 min at room temperature, and then digested for 55 s at 37°C with DNase I. The protected region of SarZ is boxed in black. **(D)** SarZ binding sequences in the *psmβ* promoter region. The SarZ binding regions, based on the DNase I footprinting analyses, are underlined in black. Putative ribosomal binding site (Shine-Dalgarno sequences, “SD”) is shaded in grey. The putative transcription start site of the *psmβ* is predicted by Softberry and denoted by asterisk (in blue, *).

Since SarZ could directly bind to the promoter region of the *psmβ* operon, we were interested in the SarZ recognition sites on the *psmβ* operon promoter. We performed DNase I footprinting analysis with the 330-bp *psmβ* operon promoter fragment labeled with 6-carboxyfluorescein (6-FAM) (**Figure 7**). Without addition of the SarZ protein, the 6-FAM labeled DNA fragment was uniformly digested, as reflected by the uniform distribution of the 6-FAM signals (**Figure 7A**). The two regions from -138 to -90 bp and -34 to -5 bp, relative to the putative transcription start site of the *psmβ* operon, were protected, as indicated by the disappearing nucleotide peaks in **Figure 7A**, compared with **Figure 7B**. These data indicated that the SarZ recognition site may lie in the 48-bp region (tcctatatgtttatatcaataaaatagagtgcaatacagttgtgcatg) and the 41-bp region (atgaaaaatgcaacaaattgagtcaaattaactttatagta) of the *psmβ* promoter.

## Discussion

*S. epidermidis* has long been considered as a harmless skin commensal and defined as a blood culture contaminant (Huebner and Goldmann, 1999). But now, with the widespread use of indwelling medical devices, it has become an important opportunistic pathogen, especially in immunocompromised patients (Kleinschmidt et al., 2015). Therefore, more attention has been attracted to the regulation of virulence in *S. epidermidis*. SarZ, belonging to the SarA family, has been well demonstrated to be the positive regulator of invasive virulence factors and negative regulator of biofilm colonization in *S. aureus*, while in *S. epidermidis*, its regulatory role seems to be exactly the opposite (Kaito et al., 2006; Wang et al., 2008; Ballal et al., 2009). However, the latter conclusion was just deduced by screening the *sarZ* transposon mutant strain in only one clinical isolate of *S. epidermidis*, and warrants further verification (Wang et al., 2008).

In this study, we successfully constructed the *sarZ* deletion mutant strain of *S. epidermidis* for the first time by allelic exchange, which can avoid the potential polar effects of transposon mutagenesis, and in another clinical isolate RP62A. Consistent with the phenotypes observed in the insertion mutant strain (Wang et al., 2008), the deletion mutant strain also exhibited significantly higher hemolytic activity and less biofilm formation, which confirmed that *SarZ* inhibits invasive virulence factors while promoting biofilm formation in *S. epidermidis*.

Traditionally, *S. epidermidis* was considered to possess no or limited hemolytic activity (Otto, 2009). Whereas, in the present study, the *sarZ* mutant strain showed remarkable hemolytic capacity, which may provide an explanation for the increased pathogenicity of *S. epidermidis* under conditions fostering opportunistic infection. Therefore, we pay much more attention to the molecular mechanism by which SarZ regulates the hemolytic activity of *S. epidermidis*. Since PSMs were known to have hemolytic activity in *S. aureus*, and in addition to harboring β-hemolysin with shingomyelinase C activity, *S. epidermidis* lacks α-hemolysin which is responsible for the zone of complete hemolysis that occurred in *S. aureus* (Peschel and Otto, 2013; Wang et al., 2007; Ahmad-Mansour et al., 2021), we thus hypothesized that SarZ might regulate the hemolytic activity by modulating the production of PSMs. As expected, the amount of PSMs indeed increased significantly in the *sarZ* mutant strain relative to the wild type strain, revealed by 1-butanol extraction and the following SDS-PAGE and hemolysis experiments.

Then, in order to determine which kinds of PSMs were affected by SarZ, the composition and content of the crude PSMs extracts was compared between the *sarZ* mutant strain and its parent strain by HPLC-MS/MS. The change in PSMs profiles was further confirmed in transcript level by qRT-PCR. As a result, it was found that the most drastically altered PSMs members were the β-type PSMs, whether in the protein expression or in the transcript level. These results prompted us to speculate that SarZ could control the hemolytic activity of *S. epidermidis* through affecting the synthesis of β-type PSMs. To test the hypothesis, the *psmβ* operon encoding the PSMβ1, PSMβ2 and PSMβ3 was knocked out in the *sarZ* mutant strain.

Although previous studies have shown that the β-type PSMs are non-cytolytic or have limited cytolytic function, and their contribution to virulence is less pronounced compared with the α-type PSMs (Peschel and Otto, 2013), the present study found that the increased hemolysis caused by *sarZ* mutation can be easily abolished by deletion of the *psmβ* operon, whereas overproduction of *psmβ* in *S. epidermidis* RP62A can lead to increase in hemolysis (**Supplementary Figure 8**), suggesting that the β-type PSMs actually have notable hemolytic effect. The discrepancy can be easily explained by the findings here and in Cheung’s laboratory that β-type PSMs are produced in a large amounts in *S. epidermidis*, accounting for almost half of the total production of PSMs, thus in sum they have an obvious impact on overall hemolytic capacity despite their hemolytic activities are much lower than, for example, that of *psmδ* or *psmα* (Cheung et al., 2010). Moreover, recent study has shown that in *Staphylococcus xylosus*, PSMβ1 and PSMα both contributed considerably to cytolysis of erythrocytes and neutrophils (Reshamwala et al., 2022). Notably, the role of *psm* genes other than *psmβ*, such as *psmα* and *psmδ*, in the SarZ-mediated regulation of hemolysis, are under investigation in our laboratory.

*Both in S. aureus* and *S. epidermidis*, expression of all *psm* genes has been reported to be under the direct control of *agr* quorum-sensing system (Cheung et al., 2014). Among the *psm* genes, *psmγ (hld*) is embedded within the RNAIII transcript, which is transcribed from P3 promoter of the *agr* system and represents the major effector molecule of the system (Queck et al., 2008; Jenul and Horswill, 2018; Tan et al., 2018). Given that the present study indicated that the expression of *psmγ (hld*) was not affected by *sarZ* mutation, and Wang’s laboratory also revealed that neither RNAIII nor other members of the *agr* system were transcriptionally altered in the *sarZ* mutant strain based on microarray data (Wang et al., 2008), it is conceivable that regulation of *psm* genes by SarZ seems to be independent of the *agr* system.These results thus let us to speculate that SarZ may directly regulate the expression of *psm* genes in *S. epidermidis.* Therefore, gel shift assay was carried out to explore whether SarZ can directly bind to the promoter regions of *psm* genes. It was found that the recombinant SarZ protein can indeed bind to the promoter regions of *psm* genes except for that of RNA III, which are in accordance to the qRT-PCR results. The DNase I footprint assay further supported that SarZ protein can bind to the promoter region of *psmβ* operon and identified the precise recognition sequences. Anyway, our data denoted that SarZ could regulate the hemolytic activity by directly controlling the expression of *psmβ* operon through its role as a transcription factor.

Biofilm is another major focus of research on the pathogenicity of *S. epidermidis* (Costerton et al., 1999). PIA is well known to constitute the main extracellular matrix of staphylococcal biofilm and synthesized by enzymes encoded by the *ica* operon (François et al., 2023). In this study, transcription of *icaA* was dramatically reduced in the *sarZ* mutant strain, suggesting that SarZ facilitates biofilm formation by activating the transcription of *ica* operon in *S. epidermidis*, which coincides with previous report (Wang et al., 2008). However, *sarZ* mutation had no obvious effect on the transcription of *icaR*, which can bind specifically to the promoter of *ica* operon to block its transcription (Conlon et al., 2002), indicating that SarZ affects *ica* operon transcription in an *icaR*-independent manner. Additionally, gel shift assay also showed that SarZ protein could directly bind to the promoter region of the *ica* operon. These observations prompted us to attempt to localize the exact SarZ-binding site on the promoter by DNase I footprinting assay (**Supplementary Figures 10A–C**). Although we failed to identify the protection region, a multiple alignment between the DNA sequence of *ica* operon promoter and the two identified SarZ recognition sequences revealed they shared high similarity in local regions, suggesting that the putative SarZ-binding motif may exist in the *ica* operon promoter (**Supplementary Figures 10D, E**). Therefore, the present study further disclosed that SarZ might directly regulate the expression of *ica* operon via its function as a transcription factor.

While most of the known winged helix proteins in bacteria such as *E.coli* MarR are repressive in nature (Grove, 2013), SarZ was demonstrated here to serve as both repressor and activator in the transcriptional regulation of downstream gene. In fact, the regulatory paradigm is universal in the SarA protein family. For instance, SarA has been found in *S. aureus* to activate *hla* (*α*−hemolysin gene) transcription but repress *spa* (protein A gene) transcription by binding to its consensus SarA recognition motif (Liu et al., 2006). However, the exact mechanism by which SarA and related family members activate and repress target genes remains unclear (Cheung et al., 2008). We proposed that co-crystallization studies with activated and repressed promoters will likely gain the molecular insights into the regulatory paradigm of SarZ and related winged helix proteins.

In summary, the present study confirmed that SarZ is a key virulence regulator of *S. epidermidis* and preliminarily resolved the underlying regulatory mechanism, namely that regulation of the hemolytic activity and biofilm formation by SarZ is achieved by modulating the transcription of *psmβ and ica* operons, respectively.

## Data Availability

The original contributions presented in the study are publicly available. This data can be found and accessed upon publication of the article here: PRIDE - Proteomics Identification Database - accession number: PXD057952.
