# Supplementary figures and images for: SarZ inhibits the hemolytic activity through regulation of phenol soluble modulins in Staphylococcus epidermidis
Source: Front Cell Infect Microbiol. 2024 Nov 19;14:1476287. doi: 10.3389/fcimb.2024.1476287 (PMC11612630; doi:10.3389/fcimb.2024.1476287)

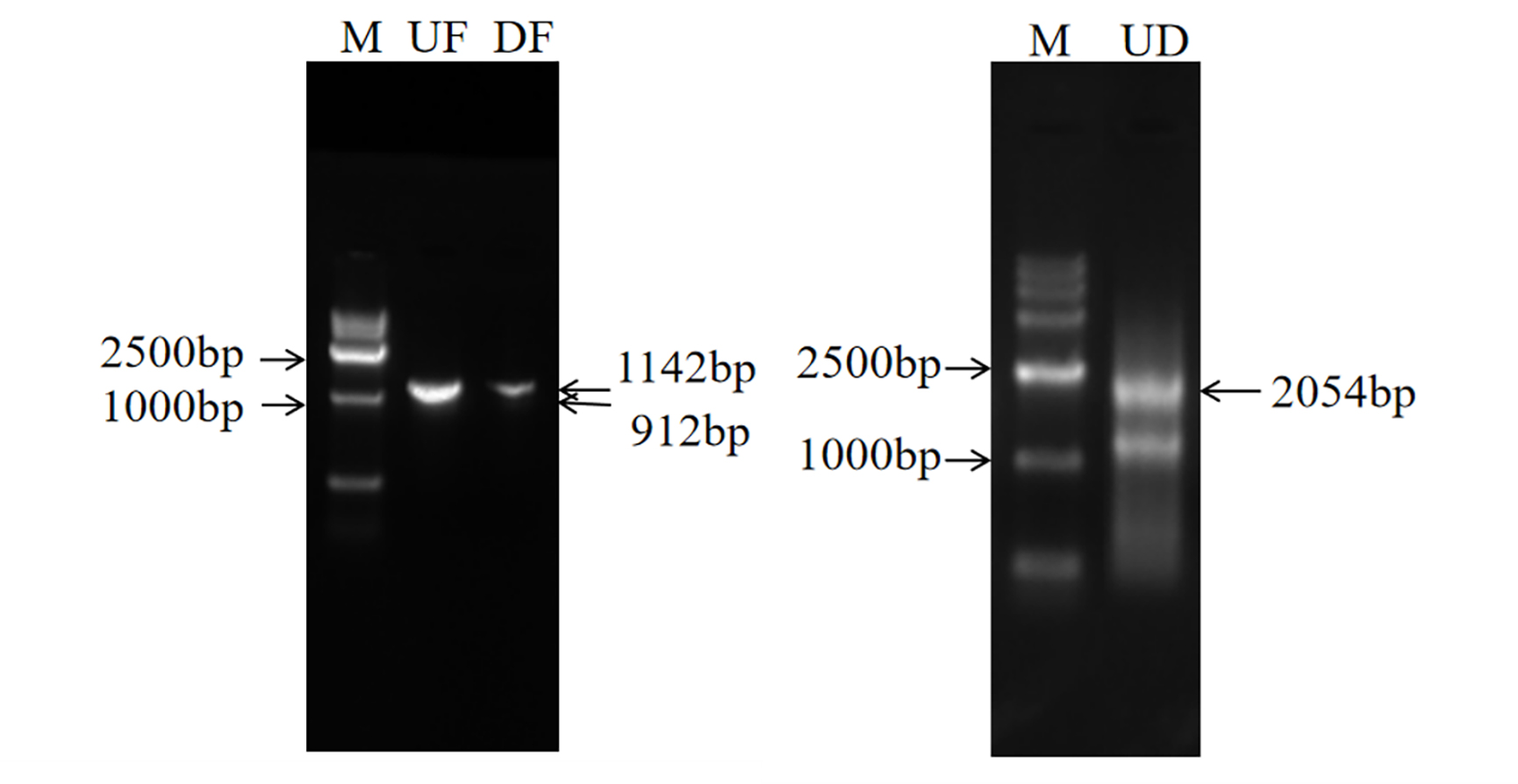

Supplement: Supplementary file 1 [file Image1.jpeg]

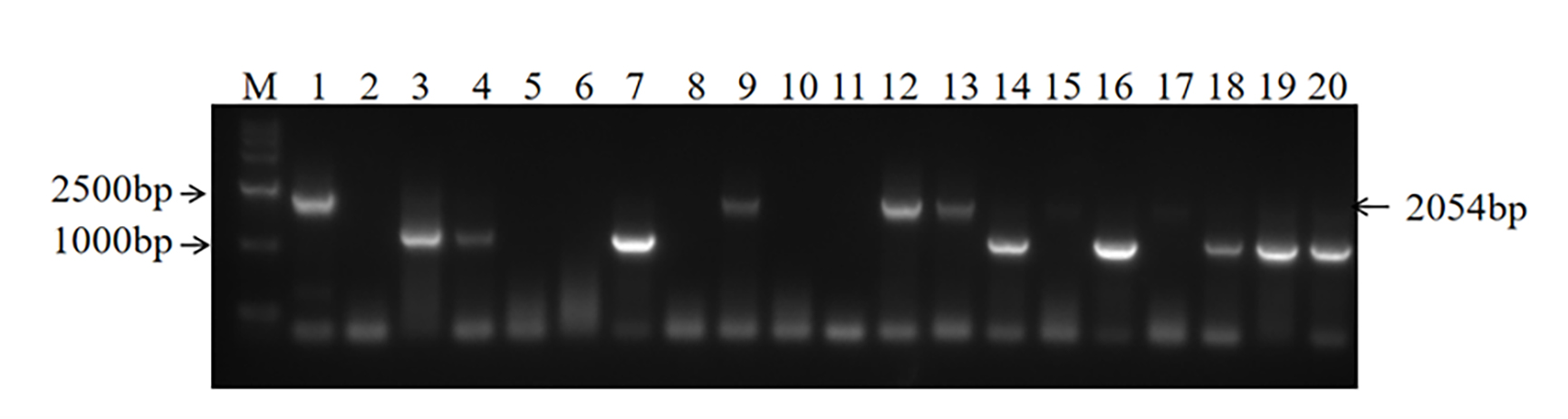

Supplement: Supplementary file 2 [file Image2.jpeg]

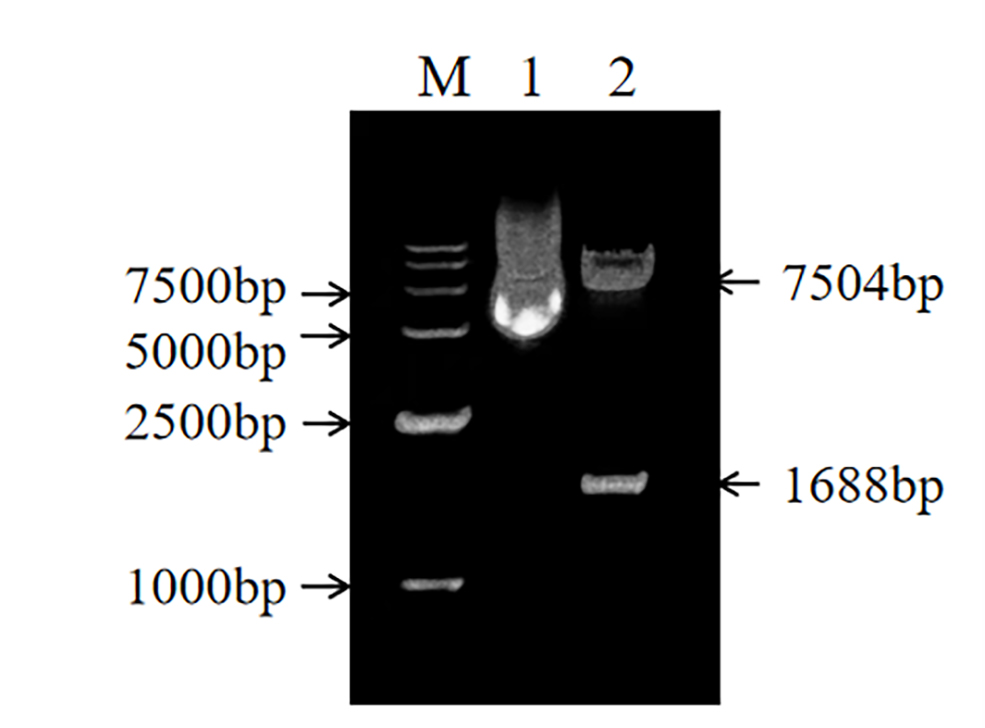

Supplement: Supplementary file 3 [file Image3.jpeg]

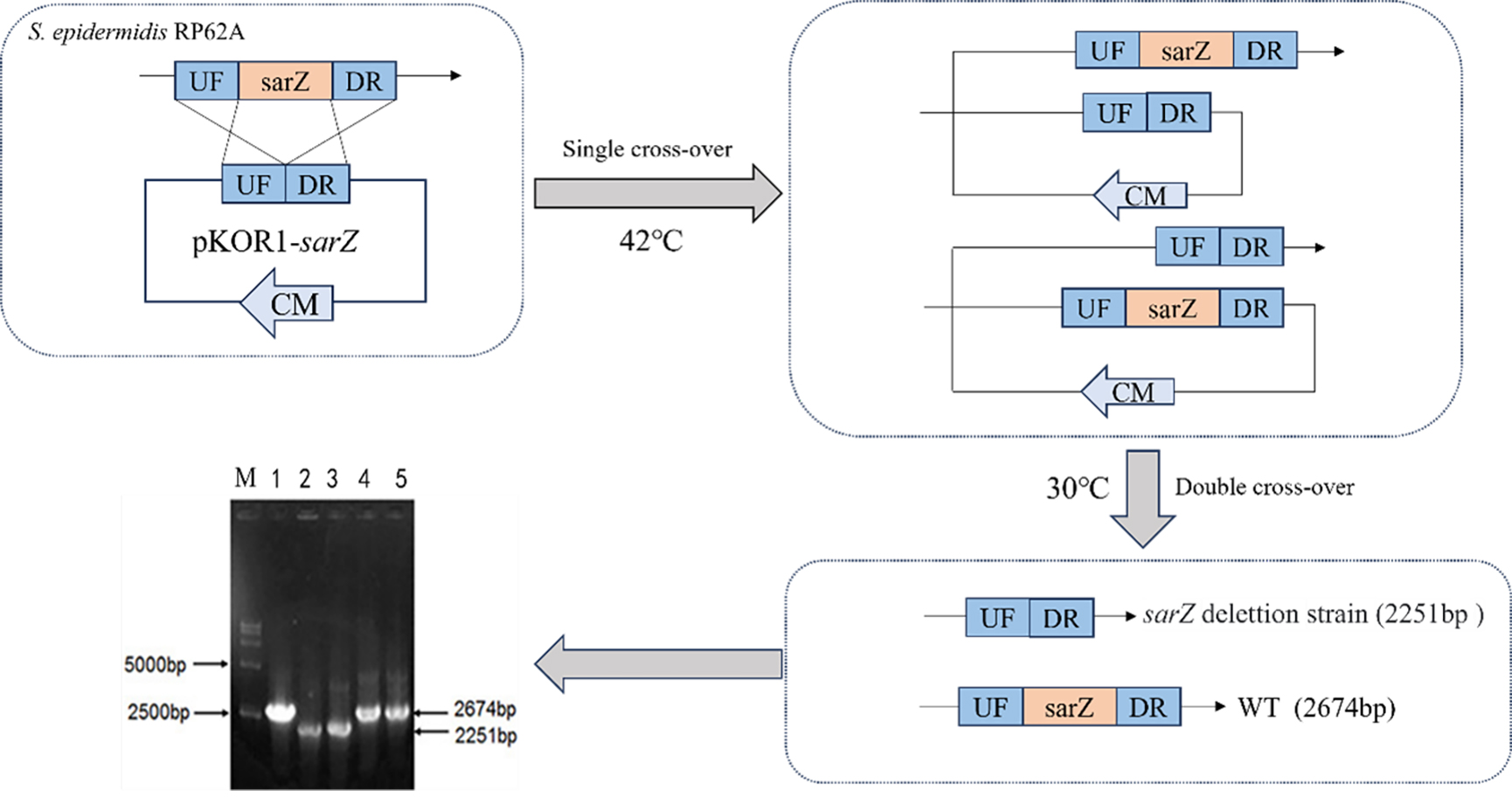

Supplement: Supplementary file 4 [file Image4.jpeg]

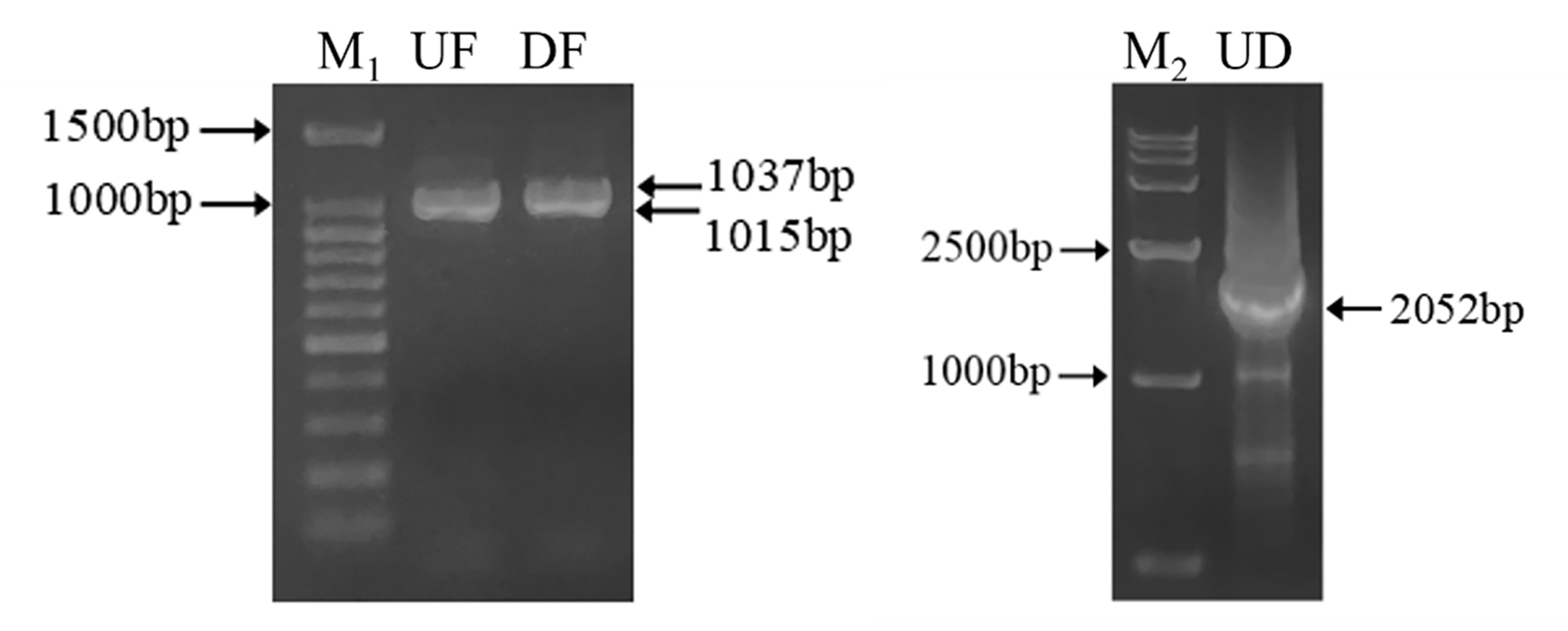

Supplement: Supplementary file 5 [file Image5.jpeg]

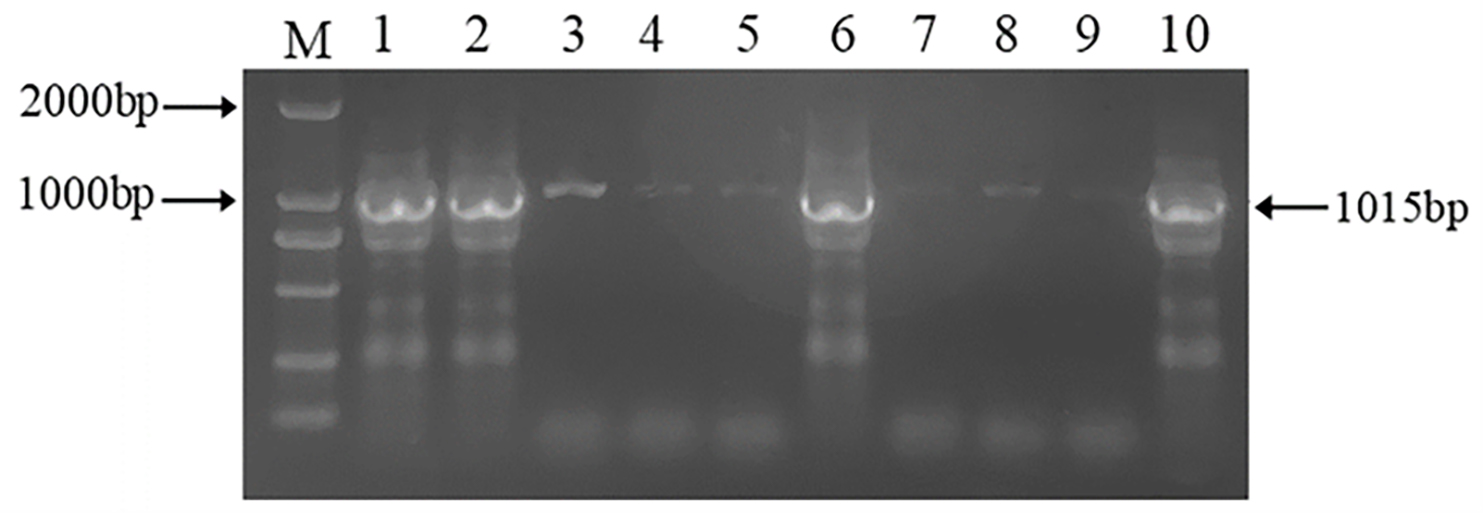

Supplement: Supplementary file 6 [file Image6.jpeg]

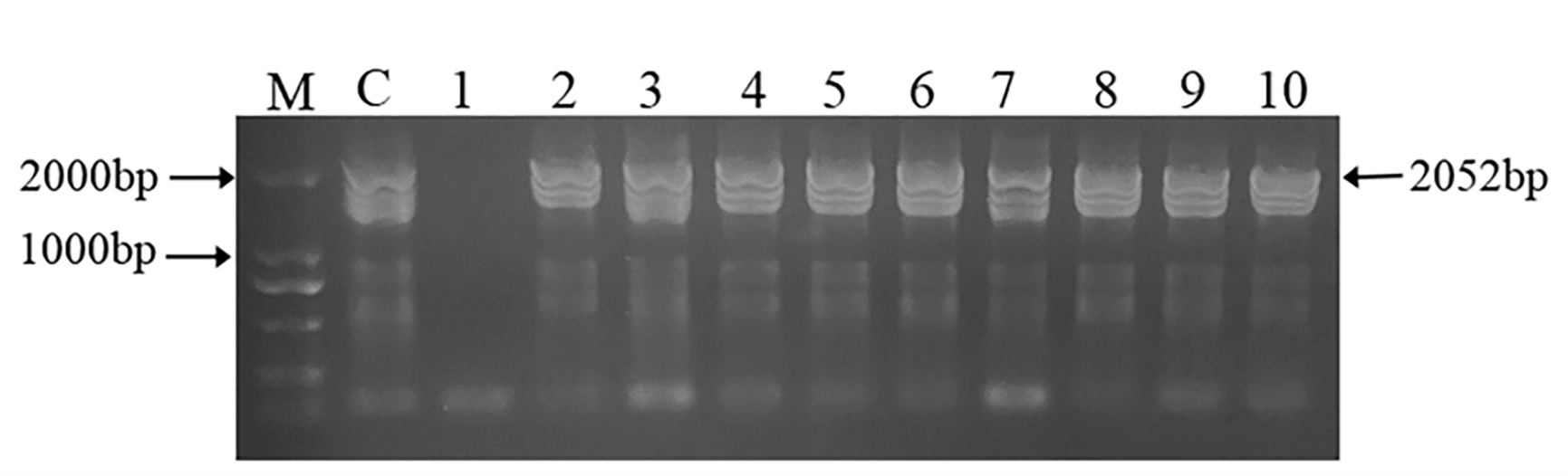

Supplement: Supplementary file 7 [file Image7.jpeg]

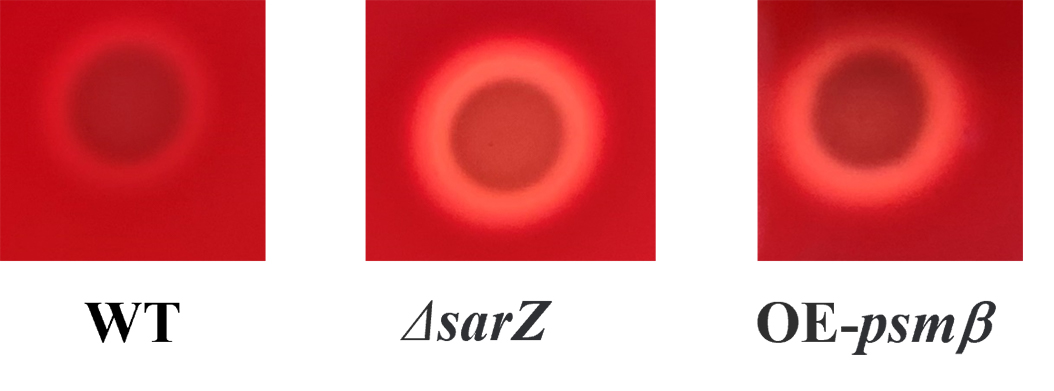

Supplement: Supplementary file 8 [file Image8.jpeg]

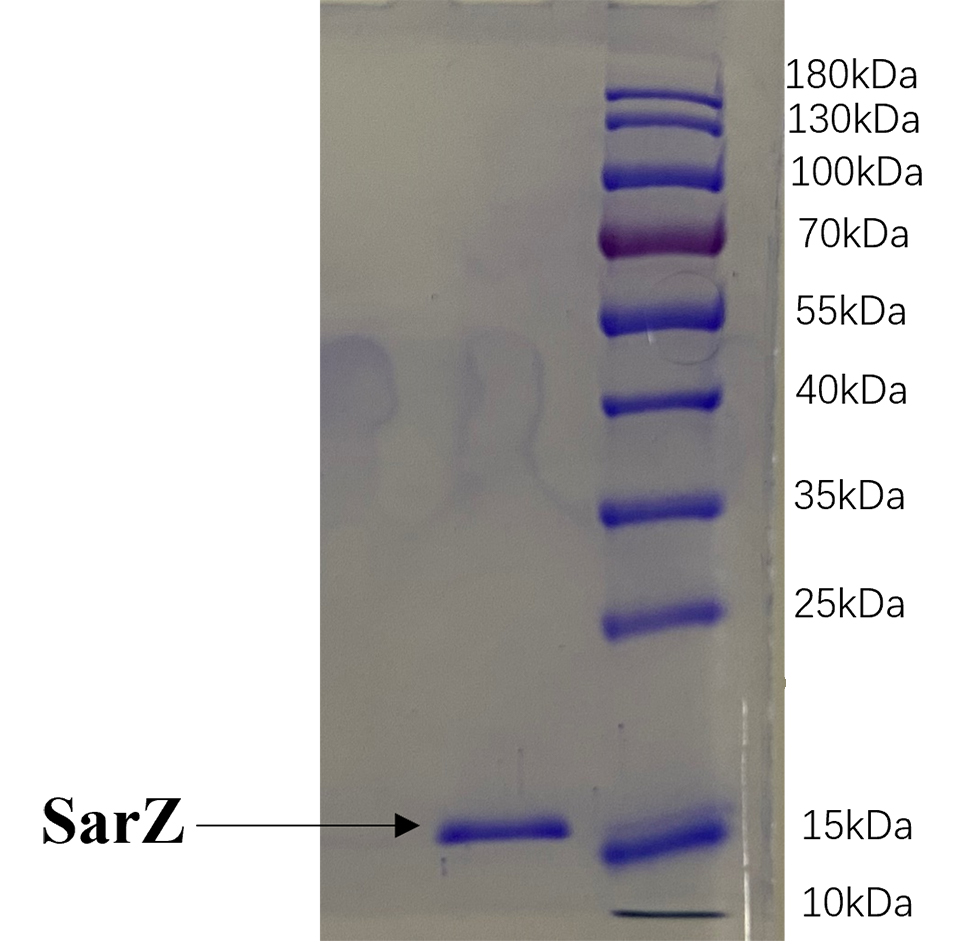

Supplement: Supplementary file 9 [file Image9.jpeg]

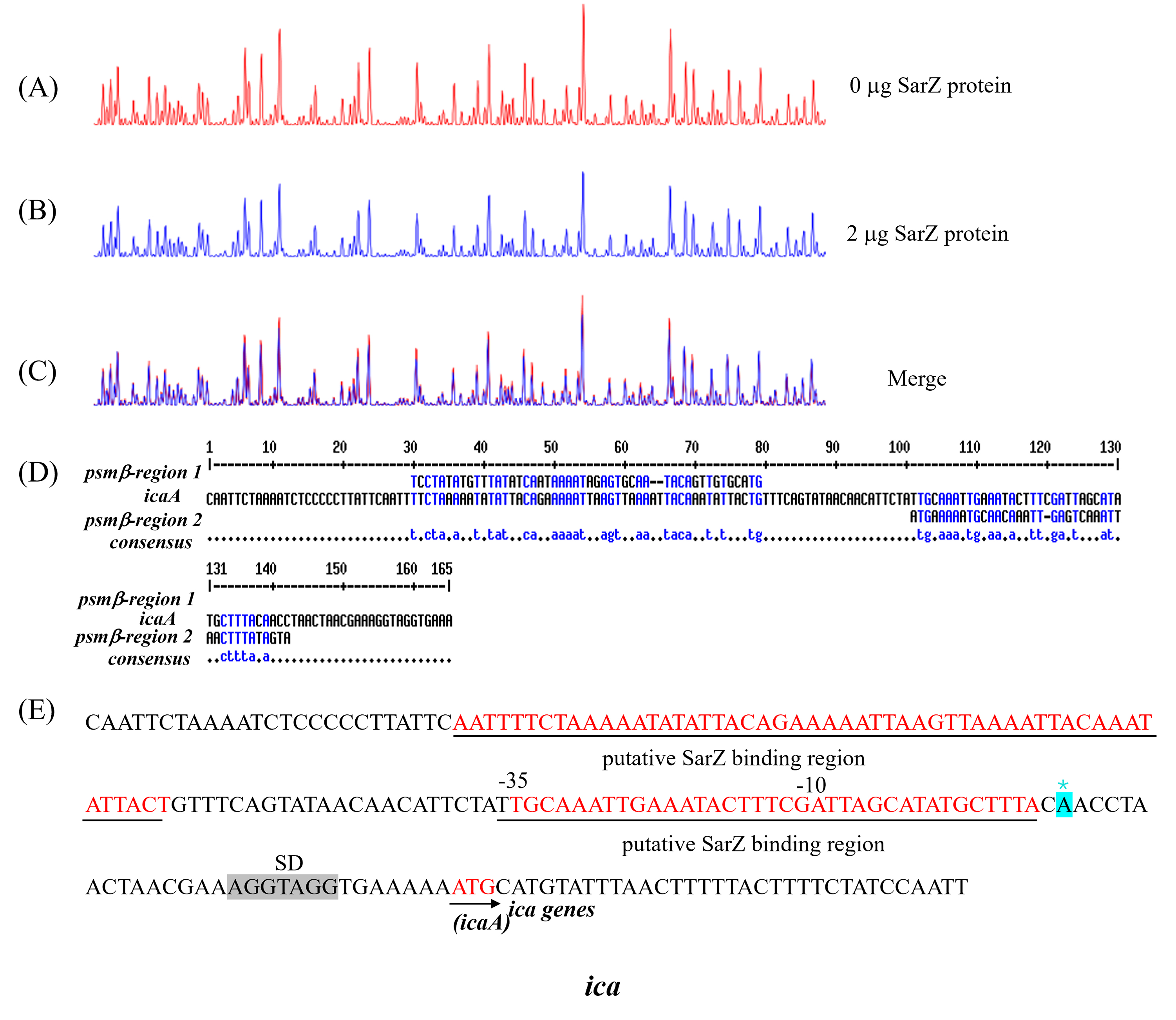

Supplement: Supplementary file 10 [file Image10.jpeg]
