# Supplementary material for: SarZ inhibits the hemolytic activity through regulation of phenol soluble modulins in Staphylococcus epidermidis
Source: Front Cell Infect Microbiol. 2024 Nov 19;14:1476287. doi: 10.3389/fcimb.2024.1476287 (PMC11612630; doi:10.3389/fcimb.2024.1476287)
Supplement: Supplementary file 11 [file Table1.docx]

Supplementary Material

# Supplementary Figure Legends and Tables

## Supplementary Figure Legends

**Supplementary Figure 1.** Amplification and Fusion of upstream and downstream fragments flanking the *sarZ* gene. The upstream and downstream fragments with the expected sizes were successfully PCR amplified (Left panel) and combined (Right panel). M: D15000 DNA marker; UF: upstream fragments. DF: downstream fragments. UD: upstream plus downstream fragments.

**Supplementary Figure 2.** Validation of the plasmid pUCm-T-*sarZ* in *E. coil* DH5α by colony PCR. M: D15000 DNA marker; Lane 1, 9, 12, 13: PCR products amplified from these colonies were consistent with the anticipated size.

**Supplementary Figure 3.** Validation of the plasmid pKOR1-*ΔsarZ* in *E. coil* DC10B by restriction endonuclease doubledigestion. M: D15000 DNA marker; Lane 1: the uncutting plasmid; Lane 2: a DNA fragment with the anticipated size was excised from the plasmid by Kpn I and Apa I.

**Supplementary Figure 4.** Diagram illustrating the construction and PCR validation of *S. epidermidis* RP62A *ΔsarZ* strain. M: D15000 DNA marker; Lane 1: *S. epidermidis* RP62A wild-type strain; Lane 2-3: the PCR products amplified from the two colonies with primers spanning the homology arms were shorter in length than that from the wild type strain (2251 bp vs 2674 bp), indicating the *sarZ* gene was successfully knocked-out in them; Lane 4-5: the *sarZ* gene was not deleted in these two colonies, since the size of the PCR product amplified from them was the same as that from the wild-type strain. UF: upstream fragments of *sarZ*. DR: downstream fragments of *sarZ*; CM: chloramphenicol resistance.

**Supplementary Figure 5.** Amplification and Fusion of upstream and downstream fragments flanking the *psmβ* gene. The upstream and downstream fragments with the expected sizes were successfully PCR amplified (Left panel) and combined (Right panel). M_1_: 100 bp DNA marker; M_2_: D15000 DNA marker; UF: upstream fragments. DF: downstream fragments. UD: upstream plus downstream fragments.

**Supplementary Figure 6.** Validation of the plasmid pUCm-T- *psmβ* in *E. coil* DH5α by colony PCR. M: D2000 DNA marker; Lane 1, 2, 6, 10: the PCR products amplified from these colonies were consistent with the expected size.

**Supplementary Figure 7.** Validation of the plasmid pKOR1-*Δpsmβ* in *E. coil* DC10B by colony PCR. M: D2000 DNA marker; C: plasmid pUCm-T-*psmβ* was used as a positive control; Lane 2-10: the amplified PCR products were consistent with the control in size, indicating the plasmid pKOR1-Δ*psmβ * successfully constructed.

**Supplementary Figure 8.** Overexpression of *psm**β* gene in *S. epidermidis* increases the hemolytic activity. The complementation plasmid pCNcat-*psmβ *was transformed into the wild-type strain RP62A to generate the *psmβ* overexpression strain OE-*psmβ*: Hemolysis assay was performed as described in Methods. The image represents one of the three independent experiments.

**Supplementary Figure 9.** Recombinant SarZ protein eluted from a Ni-column was electrophoresed on 12% SDS-PAGE and the gel was stained with Coomassie brilliant blue.

**Supplementary Figure 10.** Mapping of the SarZ recognition site in the *ica* promoter by DNase I footprinting. The promoter regions of *ica* were labeled with 6-FAM, and incubated with SarZ at 0 μg (A), 2 μg (B) for 30 min at room temperature, and then digested for 55 s at 37°C with DNase I. Based on alignment with SarZ binding sequence in the *psmb* promoter (D), putative SarZ binding region in the *ica* operon are predicted and underlined in black (E). Putative ribosomal binding site (Shine-Dalgarno sequences, “SD”) is shaded in grey. The transcription start site of the *ica* operon is denoted by asterisk (in blue, *)(Heilmann *et al.*, 1996).

Reference

Heilmann, C., Schweitzer, O., Gerke, C., Vanittanakom, N., Mack, D., and Götz, F. (1996) ‘Molecular basis of intercellular adhesion in the biofilm-forming Staphylococcus epidermidis’, *Molecular Microbiology*, 20(5), pp. 1083–1091. Available at: https://doi.org/10.1111/j.1365-2958.1996.tb02548.x.

## Supplementary Tables1. Primers used in this study ^a^

| Primer name | Sequence (5*'*-3*'*) ^b^ | Restriction enzyme | Product size (bp) |
| --- | --- | --- | --- |
| For construction and identification of *sarZ* deletion mutant | | | |
| *sarZ*-UF | GGGGACAAGTTTGTACAAAAAAGCAGGCTCGACTTCTTTATAGTATAGCCC |  | 1142 |
| *sarZ*-UR | CTCTATAAGTATCACTTTTCCACCTCTACATACATTTAATCACTCCT |  |  |
| *sarZ*-DF | AGGAGTGATTAAATGTATGTAGAGGTGGAAAAGTGATACTTATAGAG |  | 912 |
| *sarZ*-DR | GGGGACCACTTTGTACAAGAAAGCTGGGTTTGGTGGTGTTATAGGTGAT |  |  |
| *sarZ*-CEF | gccagtcttaagctcgggcccGAAGTCTCACTGAATCGTGTGCA | Apa I | 1688 |
| *sarZ*-CER | ttgagcctcggaaccggtaccGAAGCAGGTTCAGCGAATGG | Kpn I |  |
| *sarZ*-IF | CGACTTCTTTATAGTATAGCCC |  | 2674 (WT)  2251 (*∆sarZ*） |
| *sarZ*-IR | TTGGTGGTGTTATAGGTGAT |  |  |
| For construction and identification of *sarZ psmβ* deletion mutant | | | |
| *psmβ*-UF | ACAGAACTTAGTTTGTATCAGCGT |  | 1015 |
| *psmβ*-UR | GCACAAAAAAAGACCAGAGCAAACGATTGGATACGATTGCGA |  |  |
| *psmβ*-DF | TCGCAATCGTATCCAATCGTTTGCTCTGGTCTTTTTTTGTGC |  | 1037 |
| *psmβ*-DR | AAGACGTGGATATGCTAAGCA |  |  |
| *psmβ*-CEF | TTGACGTTGAGCCTCGGAACACAGAACTTAGTTTGTATCAGCGTGTG |  | 2052 |
| *psmβ*-CER | ACAGGAAACAGCTATGACATAAGACGTGGATATGCTAAGCAACTA |  |  |
| *psmβ*-IF | CAGTCATCGTGGAAATGCTAT |  | 3096 (WT)  2339 (*∆psmβ*） |
| *psmβ*-IR | GCGAGAATGGCACTACTTGA |  |  |
| pKOR1-CEF | ATGTCATAGCTGTTTCCTGTGTGAA |  | 7500 |
| pKOR1-CER | GTTCCGAGGCTCAACGTCAA |  |  |
| For complementation of *sarZ* deletion mutant | | | |
| *sarZ*-CF | gtcaatgtctgaacctgcaCTTGACAAGTGATACGCTTCATAA |  | 805 |
| *sarZ*-CR | TTGGTGGTGTTATAGGTGAT |  |  |
| CN51-CF | GGTCGACTCTAGAGGATC |  | 6389 |
| CN51-CR | TGCAGGTTCAGACATTGAC |  |  |
| Forcomplementation of *sarZ psmβ* deletion mutant | | | |
| *psmβ*-CF | caggtcgactctagaggatccaggaggATCGCAATCGTATCCAATCGT | BamH I | 1014 |
| *psmβ*-CR | agaataggcgcgcctgaattcCAATGGGCTTGGTTCACTT | EcoR I |  |
| For qRT-PCR | | | |
| *psmβ1*-F | AAGCAGCACAAGATCAAGATTGGA |  | 80 |
| *psmβ1*-R | ACCTAATACGCTAACGCCACT |  |  |
| *psmβ2*-F | TTTGACGCAATTAGAAGTG |  | 88 |
| *psmβ2*-R | CATTTTCAACAATACCAGC |  |  |
| *psmβ3*-F | TAGAAGCAGCCATCACTAACGA |  | 86 |
| *psmβ3*-R | TCGATTCACCATATCAACGCTACTT |  |  |
| *psmα*-F | TTGATCAATTAAGCCTTTAACAA |  | 53 |
| *psmα*-R | TGGCAGATGTAATCGCTAAAA |  |  |
| *psmδ*-F | aacgatgtctacgatagttttaac |  | 54 |
| *psmδ*-R | atgagcatcgtatcaactatcat |  |  |
| *icaA*-F | ACGAACCACGTGCTCTATGC |  | 82 |
| *icaA*-R | CCTTGAGCCCATCGAACCCT |  |  |
| *icaR*-F | AAACTGGTAAAGTCCGTCAATGG |  | 200 |
| *icaR*-R | TTTCCGAAAAGGGGTACGATGG |  |  |
| *psme*-F | TCAACGTCGTACAATCAAT |  | 171 |
| *psme*-R | AACAAATACATAGGAGGTG |  |  |
| *psmg*-F | GGCTCAACAACTCACTAAT |  | 143 |
| *psmg*-R | ATCATTTCTACAATCGGTG |  |  |
| *gyrB*-F | CACCGTGAAGACCGCCAGATAC |  | 99 |
| *gyrB*-R | AGATGGGACGCCCTGCTGTC |  |  |
| For recombinant expression of SarZ | | | |
| pET-28a-*sarZ*-F | ggaattccatatgGAGAATAGTTATTTGAGCAAAC | Nde I | 438 |
| pET-28a-*sarZ*-R | ccgctcgagtcaCTTTTCCACTGTTTTATCA | Xho I |  |
| For amplification of promoter fragments in EMSA assay | | | |
| P*_psmβ_*-F | cttttagcaataggtacc |  | 276 |
| P*_psmβ_*-F-biotin | cttttagcaataggtacc |  |  |
| P*_psmβ_*-R | ttggatacgattgcgattc |  |  |
| P*_ica_*-F | CAATTCTAAAATCTCCCC |  | 165 |
| P*_ica_*-F-biotin | CAATTCTAAAATCTCCCC |  |  |
| P*_ica_*-R | TTTCACCTACCTTTCGTTA |  |  |
| P*_psma_*-F | gcaagacttagttattacc |  | 262 |
| P*_psma_*-F-biotin | gcaagacttagttattacc |  |  |
| P*_psma_*-R | gtgcatagtccctaaatta |  |  |
| P*_psme_*-F | cccagttttatctttatg |  | 230 |
| P*_psme_*-F-biotin | cccagttttatctttatg |  |  |
| P*_psme_*-R | taaactattctctactccg |  |  |
| P*_psmg_*-F | tataacttcactcctttcgaattaaggtaa |  | 183 |
| P*_psmg_*-F-biotin | tataacttcactcctttcgaattaaggtaa |  |  |
| P*_psmg_*-R | acagttgagtactaaatattgctatttacg |  |  |
| P*_rpsJ_*-F | AAGATTCTCGTGAACAATTC |  | 119 |
| P*_rpsJ_*-F-biotin | AAGATTCTCGTGAACAATTC |  |  |
| P*_rpsJ_*-R | GATGTCTACACCTGATGG |  |  |
| For amplification of promoter fragments in DNase I Footpriting assay ^c^ | | | |
| P-F-FAM | GTTGTAAAACGACGGCCAG |  | *psmβ*:330 bp  *ica*:254 bp |
| P-R | CAGGAAACAGCTATGAC |  |  |

# ^a^ The primers were designed using Primer Premier 5 software according to the genomic sequence of *S. epidermidis* RP62A.

# ^b^ Restriction sites are indicated by underlining.

# ^c^ The primers used to amplify the target fragments on the plasmid were pUC57 universal primers.
